# Elevated levels of TRF2 induce telomeric ultrafine anaphase bridges and rapid telomere deletions

**DOI:** 10.1038/ncomms10132

**Published:** 2015-12-07

**Authors:** Bernadette Nera, Hui-Shun Huang, Thao Lai, Lifeng Xu

**Affiliations:** 1 Department of Microbiology and Molecular Genetics, University of California, Davis, California 95616, USA

## Abstract

The shelterin protein TRF2 is essential for chromosome-end protection. Depletion of TRF2 causes chromosome end-to-end fusions, initiating genomic instability that can be cancer promoting. Paradoxically, significant increased levels of TRF2 are observed in a subset of human cancers. Experimental overexpression of TRF2 has also been shown to induce telomere shortening, through an unknown mechanism. Here we report that TRF2 overexpression results in replication stalling in duplex telomeric repeat tracts and the subsequent formation of telomeric ultrafine anaphase bridges (UFBs), ultimately leading to stochastic loss of telomeric sequences. These TRF2 overexpression-induced telomere deletions generate chromosome fusions resembling those detected in human cancers and in mammalian cells containing critically shortened telomeres. Therefore, our findings have uncovered a second pathway by which altered TRF2 protein levels can induce end-to-end fusions. The observations also provide mechanistic insight into the molecular basis of genomic instability in tumour cells containing significantly increased TRF2 levels.

## Abstract (web summary)

TRF2 is a member of the shelterin complex, essential in protecting telomeres from end-to-end fusions; however, increased levels of TRF2 are associated with cancer. Here, the authors show overexpression of TRF2 results in genomic instability through replication fork stalling and chromosome fusions.

## Graphical abstract

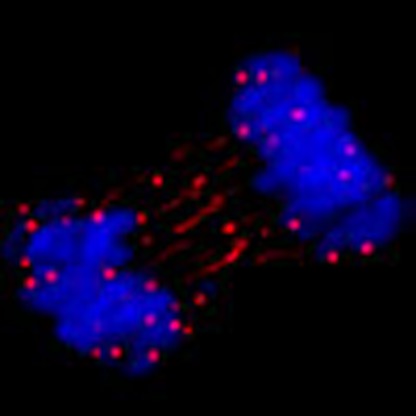

Shelterin—a six-protein complex bound to chromosome termini—is essential for protecting the integrity of natural chromosome ends^1^. Within the shelterin complex, POT1 binds single-stranded telomeric overhang^2^, while TRF1 and TRF2 bind duplex telomeric DNA^3^,^4^,^5^ and recruit TIN2, TPP1, POT1 and Rap1 to telomeres through protein–protein interactions^6^,^7^,^8^,^9^,^10^,^11^. When shelterin proteins are experimentally depleted, telomeres are sensed by cells as aberrant DNA. This triggers DNA damage signalling at telomeres, resulting in inappropriate repair, which can produce chromosome end-to-end fusions.

Shelterin is also involved in regulating the length of the telomeric tract in addition to chromosome end protection. The most recognized pathway is through its dual role in the regulation of telomerase: The shelterin component TPP1 promotes telomerase function by recruiting telomerase to telomeres via a direct interaction between its N-terminal OB-fold domain and the telomerase catalytic subunit^12^,^13^,^14^,^15^,^16^. Mutations that disrupt this interaction compromise telomerase-dependent telomere elongation. In contrast, the shelterin component TRF1 is thought to block telomerase access to telomeres through anchoring POT1 to telomeres^17^. Overexpression of TRF1 results in gradual telomere shortening^18^,^19^ and epistasis experiments have demonstrated that this effect is through inhibition of telomerase activity^20^.

However, telomere length homeostasis is dictated by more than simply telomerase action. In young primary human somatic cells, occasionally extremely shortened telomeres can be detected well before senescence^21^. Ultrashort telomeres (named ‘t-stumps') of sizes significantly different from the bulk telomere size distribution also exist in cancer cells, which contain active telomerase^22^. It is speculated that such ultrashort telomeres in primary or cancer cells are generated through stochastic loss of long tracts of telomeric repeats^21^, a process that is different from the progressive telomere loss caused by replicative attrition due to lack of telomerase. Notably, the shelterin protein TRF2 has been reported to trigger telomere shortening by an unknown mechanism in a telomerase-independent manner^19^,^20^,^23^,^24^: overexpression of TRF2 can accelerate the rate of telomere erosion in human primary cells that do not have telomerase^20^,^24^, and even trigger a DNA damage response^25^. This suggests that TRF2 is involved in a telomere-processing function that is different from telomerase inhibition.

Purified shelterin components have also been reported to stall replication fork progression at telomeric sequences in an *in vitro* SV40 DNA-based replication system^26^, suggesting another mechanism by which telomere length might be modulated. Unresolved DNA structures during replication can persist through mitosis and cause the formation of ultrafine anaphase bridges (UFBs)^27^,^28^,^29^,^30^,^31^. Unlike canonical anaphase bridges that originate from covalent chromosome fusions, UFBs arise from interlinked sister chromatids. Two different types of UFBs have been described: one type of UFB forms at centromeres and likely derives from fully replicated DNA sequences held together by DNA catenation. They can be induced by topoisomerase II inhibitors^27^,^30^,^31^. The second type of UFB, which usually associates with common fragile sites (CFS), presumably derives from incompletely replicated DNA sequences and can be exacerbated by replication inhibitors^28^,^29^. Mammalian telomeres have been suggested to resemble CFS^32^, appearing as decondensed or multiple split signals in metaphase chromosomes under replication stress. Ultrafine anaphase bridges that are composed of telomeric sequences, however, are extremely rare, even when cells were challenged with replication inhibitors^28^.

In this study, we have asked whether elevated levels of TRF2 might promote the pathway that gives rise to ultrashort telomeres. We show here that TRF2 overexpression in human cells stalls replication at telomeric sequences and induces the formation of thin threads of telomeric bridges that arise during segregation of anaphase chromosomes. The induction of these telomeric UFBs precedes stochastic loss of large segments of telomeric sequences, with a subsequent increase in chromosome fusions. Since significantly elevated levels of TRF2 have been detected in many tumour samples and cancer cell lines^23^,^33^,^34^,^35^, as well as during the transformation of human primary mammary epithelial cells^36^, our findings provide mechanistic insight into a specific molecular mechanism driving genome instability in tumour cells.

## Results

### Elevated levels of TRF2 cause stochastic telomere shortening

**Figure 1:**
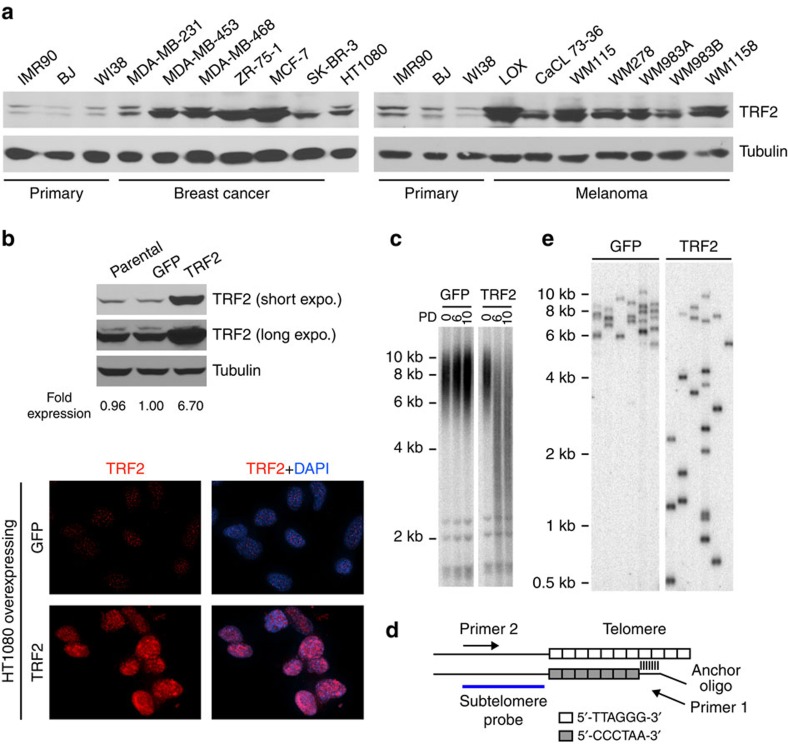
TRF2 overexpression in HT1080 cells led to stochastic shortening of telomeres. (**a**) Elevated levels of TRF2 protein in a number of breast cancer and melanoma cells. Immunoblotting was performed to detect TRF2 in whole-cell extracts of the following human cell lines: Primary fibroblasts: IMR90, BJ and WI38; Breast cancer cells: MDA-MB-231, MDA-MB-453, MDA-MB-468, ZR-75-1, MCF-7 and SK-BR-3; Melanoma cells: Lox, CaCL 73-36, WM115, WM278, WM983A, WM983B and WM1158. Fibrosarcoma cell: HT1080. Tubulin was used as a loading control. (**b**) Assessing TRF2 overexpression levels. Parallel cultures of HT1080 clone A6 (a subclone of HT1080 cells that maintain stable telomere length) cells infected with lentiviruses expressing GFP or TRF2 were examined by immunoblotting (top panel) or immunostaining (bottom panel). Fold of TRF2 expression was quantified by the ImageJ software and normalized to tubulin levels. (**c**) Terminal Restriction Fragment analysis of HT1080 A6 cells infected with lentiviruses expressing GFP or TRF2. Cells were continuously passaged and collected at the indicated population doublings (PD). (**d**) Schematic diagram of STELA analysis. (**e**) Individual telomere lengths measured by STELA analysis in HT1080 A6 cells overexpressing GFP or TRF2 at PD6. Each lane represents a single PCR reaction performed with 100 pg of genomic DNA, followed by Southern blotting detection of XpYp telomeres using an XpYp subtelomeric probe.

We carried out western blotting analysis to examine TRF2 protein expression in multiple human melanoma, breast cancer and primary cell lines. TRF2 was found to be expressed at significantly higher levels (approximately two- to eightfold) in several breast cancer (MDA-MB-453, MDA-MB-468, ZR-75-1 and MCF-7) and melanoma cell lines (LOX, WM115, WM278, WM983A and WM1158) compared with the primary cells (IMR90, BJ and WI38) (Fig. 1a, Supplementary Fig. 1a), consistent with observations reported by other groups^23^,^33^,^34^,^35^.

To understand how elevated TRF2 levels might affect telomere maintenance, we overexpressed full-length, untagged wild-type TRF2 (Fig. 1b) in HT1080 human fibrosarcoma cells to approximately sevenfold of endogenous level using a lentiviral expression system and analysed the effects on telomeres. HT1080 cells were chosen for this study because their endogenous TRF2 level is comparable to that in primary cells (Fig. 1a), and they have active telomerase (Supplementary Fig. 1b), which maintains telomeres at a stable intermediate-length range. As shown in Fig. 1c, within six population doublings (PDs), the distribution of bulk telomeres in cells overexpressing TRF2 changed from a tight cluster between 6 and 10 kb to a smear that extended from ∼10 kb to below 2 kb; bulk telomeres in control cells overexpressing GFP maintained stable lengths, as expected.

To investigate this effect at a higher resolution, we used Single Telomere Length Analysis (STELA), which examines the length of individual telomeres^21^. In this assay (depicted in Fig. 1d), an anchor oligonucleotide comprising a unique sequence of 20 bases followed by 7 bases of telomeric repeat homology is ligated to the 5′-end of the C-rich strand of telomeric DNA. After ligation, the genomic DNA is diluted and different aliquots that each contain a small population of telomeres are analysed by PCR with the indicated primers. Southern hybridization to a probe containing the subtelomeric sequence of a specific chromosome (for example, a sequence at the end of the common subtelomeric sequence on the short arms of X and Y chromosomes, XpYp) is then used to detect the PCR products. Individual bands in the STELA analysis therefore represent the double-stranded region of a single telomere (also containing a short defined subtelomeric sequence). As shown in Fig. 1e, the majority of STELA products of XpYp telomeres in HT1080 cells overexpressing GFP ranged between 6 and 10 kb, consistent with the bulk telomere length results shown in Fig. 1c. In contrast, in cells overexpressing TRF2, the STELA products were very heterogeneous, with sizes ranging between 0.5 and 10 kb. This argues that TRF2 overexpression resulted in stochastic telomere shortening events that occurred within a very limited number of cell divisions.

**Figure 2:**
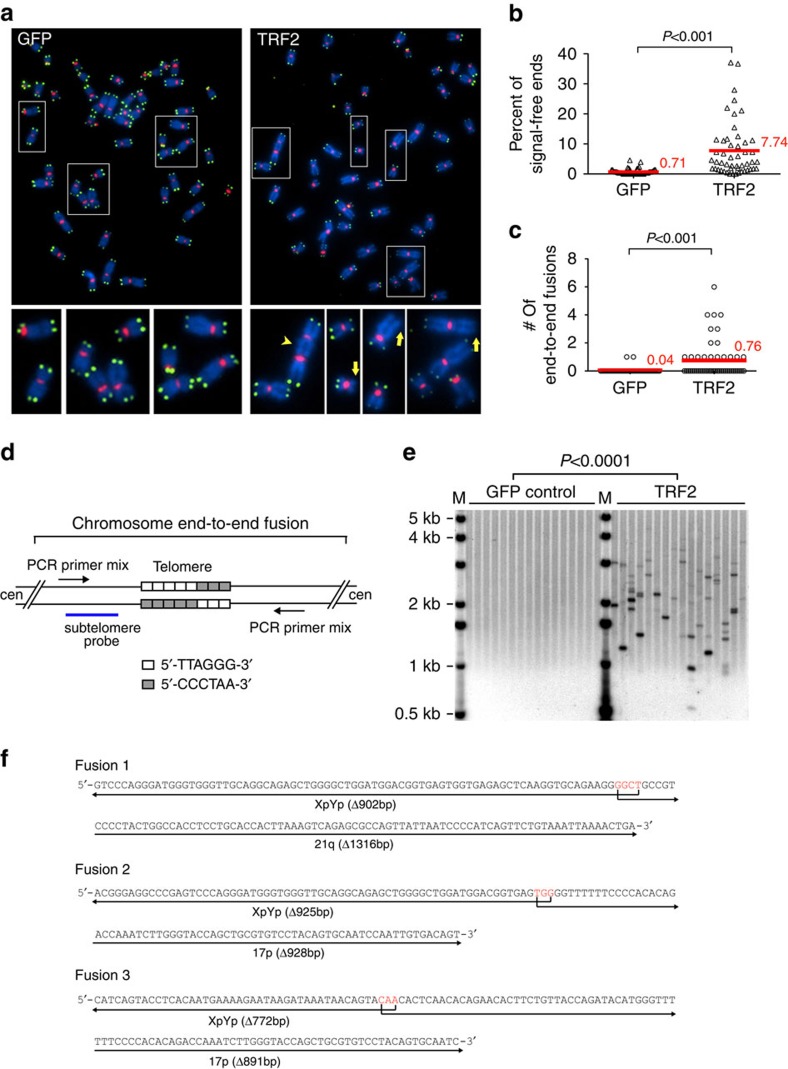
Infrequent chromosome end-to-end fusions in HeLa1.2.11 cells overexpressing TRF2. (**a**) Representative metaphase spread image of HeLa1.2.11 cells infected with lentivirus expressing GFP or TRF2. Infected cells were passaged and collected at PD7 for metaphase spread followed by FISH analysis. Chromosomes (blue) were hybridized with PNA probes for telomeric sequences (green) or centromeric sequences (red). Regions in white boxes are enlarged to the bottom of the corresponding image for better visualization. Yellow arrows indicate signal-free telomeres; arrowhead indicates chromosome end-to-end fusions. For **b** and **c**, 50 metaphases (∼3,360 chromosomes) each of GFP- or TRF2-overexpressing cells were examined for telomeric abnormality. All quantifications were carried out blindly. Each point on the scatter plot represents a single metaphase. Mean values are indicated in red. Two-tailed Student's *t*-tests were performed to make pairwise comparison for statistical significance. (**b**) Quantification of signal-free telomeres in HeLa1.2.11 cells overexpressing GFP or TRF2. (**c**) Quantification of chromosome end-to-end fusions in HeLa1.2.11 cells overexpressing GFP or TRF2. (**d**) Schematic diagram of Fusion PCR analysis. (**e**) Individual chromosome end-to-end fusions assessed by Fusion PCR. HeLa1.2.11 cells overexpressing GFP or TRF2 were harvested at PD6. Multiple aliquots of 100 ng of genomic DNA were independently subjected to fusion PCR using a mix of XpYp, 17p and 21q subtelomeric primers. PCR products were resolved on 1% agarose-TBE gel and detected by Southern hybridization with an XpYp-specific subtelomeric probe. (**f**) Representative sequence of fusion molecules between XpYp, 17p and 21q. The fusion points, size of deletion, and microhomology (in red) are indicated.

Notably, we detected STELA products as short as ∼0.5 to 0.6 kb. Considering that the STELA products of XpYp telomeres contain ∼0.4 kb of subtelomeric region, these results indicate that TRF2 overexpression can infrequently lead to the loss of almost the entire telomeric tract of some chromosomes, potentially causing chromosome end deprotection. To test whether this was indeed the case, we examined telomere morphology by performing fluorescence *in situ* hybridization (FISH) with a telomeric repeat probe on metaphase chromosomes. HeLa1.2.11 cells were used for this assay because their long telomeres (mean telomere lengths ∼20 kb) provide strong and easily detectable fluorescence signal for the FISH-based detection of telomeres (Fig. 2a). Cells were collected seven population doublings (PD7) after TRF2 overexpression for this analysis. We observed a statistically significant increase in signal-free chromosome ends (Fig. 2b) and chromosome end-to-end fusions (Fig. 2c) in cells overexpressing TRF2. Strikingly, the majority of TRF2-induced chromosome fusions lacked detectable telomeric signals at the fusion junction (Fig. 2a), suggesting that the loss of telomeric sequences precedes the chromosome fusion events.

We further examined these TRF2-induced chromosome fusions using a PCR-based assay^37^ (Fig. 2d). In this assay, individual chromosome fusions were first amplified by PCR utilizing telomere-proximal subtelomeric oligonucleotide primers of selected chromosomes, and then detected by Southern hybridization with a subtelomeric probe. Once again, we observed a significant increase of chromosome fusions in HeLa1.2.11 cells overexpressing TRF2 with this molecular assay (Fig. 2e), consistent with the FISH data. Sequencing of the amplified fusion products revealed that all fusions involved chromosome ends that completely lacked telomeric repeat DNA, with deletions often extending well into the telomere-adjacent subtelomeric tracts (Fig. 2f, Supplementary Fig. 2). All of the fusion molecules sequenced contained unique deletion points at the fusion junction, reflecting the stochastic nature of the deletion events. Most of the fusions also had one to six nucleotides of microhomology between the fused chromosomes at the fusion points (Fig. 2f, Supplementary Fig. 2). Similar large deletions and microhomologies have been observed in chromosome fusions detected in mammalian cells containing short dysfunctional telomeres and in early-stage colon carcinoma and chronical lymphocytic leukaemia cells^37^,^38^,^39^,^40^,^41^.

Collectively, the above observations indicate that in response to elevated levels of TRF2, infrequently a fraction of chromosome termini are critically shortened, which leads to a loss of end protection and subsequent end-to-end fusions.

### Stalled telomere replication and telomeric UFB formation

**Figure 3:**
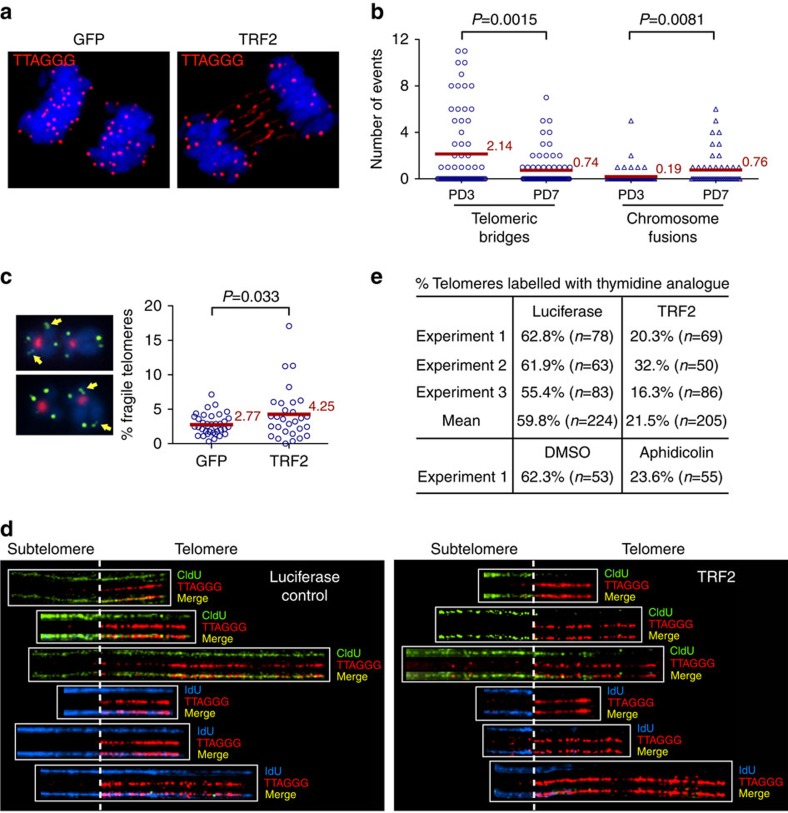
TRF2 overexpression induced telomeric ultrafine anaphase bridges. (**a**) Formation of thinly stretched telomere bridges between anaphase chromosomes in HeLa1.2.11 cells overexpressing TRF2. Telomeric DNAs were detected by *in situ* hybridization with a PNA telomeric probe (red). Chromosomes were stained with DAPI (blue). (**b**) Quantification of telomeric anaphase bridges and chromosome end-to-end fusions in HeLa1.2.11 cells overexpressing TRF2 at PD3 and PD7. HeLa1.2.11 cells were infected with lentiviruses expressing TRF2. Parallel cultures were collected at PD3 or PD7 for PNA telomere-FISH to examine telomeric anaphase bridges or for metaphase spreading followed by PNA telomere-FISH to examine chromosome end-to-end fusions. (**c**) Quantification of fragile telomeres in HeLa1.2.11 cells overexpressing GFP control or TRF2 at PD3. Cells were collected for metaphase spreading followed by PNA telomere-FISH to examine fragile telomeres. Representative fragile telomeres are labelled by yellow arrows on images at the left panel. All quantifications were carried out blindly. For **b** and **c**, mean values are indicated in red. Two-tailed Student's *t*-tests were performed to make pairwise comparison for statistical significance. (**d**) TRF2 overexpression stalled replication at telomeres. Representative chromatin fibre FISH images showed the incorporation of IdU (blue) or CldU (green) at telomeric (red) and adjacent subtelomeric regions in LOX cells infected with lentiviruses expressing luciferase control or TRF2. At PD3, cells in logarithmic growth were labelled sequentially with 30 μM of IdU and then CldU for 4 h each before Chromatin fibre-FISH analysis was carried out. Telomeres were identified by FISH with a telomeric repeat probe. IdU and CldU were identified by immunostaining with analogue-specific antibodies. Dotted line represents the start of telomeric sequences. We did not see dual IdU and CldU labelling at replicating telomeres due to the long labelling time (4 h) used for each halogenated nucleotide. (**e**) Quantification of fraction of telomeric fragments that was labelled with CldU and/or IdU. For control of stalled replication, cells were treated with 1 μg ml^−1^ aphidicolin for 16 h before they were labelled with IdU and CldU in the presence of aphidicolin (see Supplementary Fig. 3c for representative images).

Analysis of anaphase chromosomes provided insight into the molecular basis for TRF2-induced stochastic telomere shortening: we observed numerous telomeric bridges, identified by a telomeric repeat PNA FISH probe, between the segregating anaphase chromosomes in different cell lines overexpressing TRF2 (Fig. 3a, Supplementary Fig. 3a). These telomeric anaphase bridges were detected as early as 24 h after the cells were infected with lentivirus expressing TRF2, before a significant amount of TRF2-induced telomere shortening became detectable (Supplementary Fig. 3b). The fine thread-like telomeric bridges were visible through FISH with a telomeric repeat probe, but not through DAPI staining.

Quantification of both chromosome fusions and telomeric anaphase bridges in HeLa1.2.11 cells overexpressing TRF2 at PD3 and PD7 showed that the number of telomeric anaphase bridges decreased from 2.14 to 0.74 per cell, while that of chromosome fusions increased from 0.19 to 0.76 per cell (Fig. 3b). These data, together with the fact that TRF2-induced chromosome fusions lacked telomeric repeat tracts (Fig. 2a,f), support the conclusion that the TRF2-induced telomeric anaphase bridges were unlikely to originate from chromosome fusions. Instead, the TRF2-induced telomeric bridges were reminiscent of the UFBs, which derive from either catenated sister chromatids or incompletely replicated DNA during mitosis^27^,^28^,^29^,^30^,^31^. We also observed that overexpression of TRF2 increased the frequency of fragile telomeres (Fig. 3c), which are aberrant decondensed and multiple split telomere signals whose formation closely correlates with replication stalling at telomeric regions^32^. This suggested that TRF2 overexpression was causing telomere replication stalling and the subsequent formation of telomeric UFBs. To assess this, we examined telomere replication by performing Chromatin Fibre-FISH analysis^32^,^42^,^43^,^44^. We used human LOX melanoma cells for this analysis because their very long telomeres (mean telomere length ∼50 kb) allow better linear resolution. Briefly, replicating DNA in LOX cells overexpressing a luciferase control or the TRF2 protein were labelled consecutively with halogenated nucleotides IdU and CldU before the cells were lysed and the chromatin fibres stretched onto a positively charged glass slide. Immunostaining was then carried out with antibodies against IdU and CldU, followed by FISH analysis with a telomeric repeat probe. The replication status of telomeres was determined by analysing the incorporation of halogenated nucleotides within telomeres.

We adopted a previously established pulse-labelling procedure for human cells, which incubates proliferating cells sequentially in IdU and CldU for 4 h each^43^. As the replication fork generally progresses at ∼2 kb min^−1^ in mammalian cells^45^, all of the halogenated nucleotide-incorporating telomeres in cells overexpressing luciferase control were completely labelled with either IdU or CldU (Fig. 3d). In cells overexpressing TRF2, many telomeres were only partially labelled or not labelled at all, even though their adjacent subtelomeric tracts were fully labelled with IdU or CldU, indicating that the replication forks stalled specifically at telomeric repeat tracts. We did not observe any telomeric tracts containing IdU segment flanked by a CldU segment on either side (Fig. 3d), suggesting that the stalled telomere replication forks failed to restart during the 4 h of CldU labelling. Quantification showed that the overexpression of TRF2 resulted in approximately threefold decrease of replicated telomeres (Fig. 3e). The extent of telomere replication stalling induced by TRF2 was comparable to that induced by 1 μg ml^−1^ of DNA polymerase inhibitor aphidicolin (Fig. 3e, Supplementary Fig. 3c). Aphidicolin-treated cells, however, failed to replicate through both the subtelomeric region and the telomeric region. This suggests that aphidicolin does not specifically stall replication forks at telomeres (Supplementary Fig. 3c).

**Figure 4:**
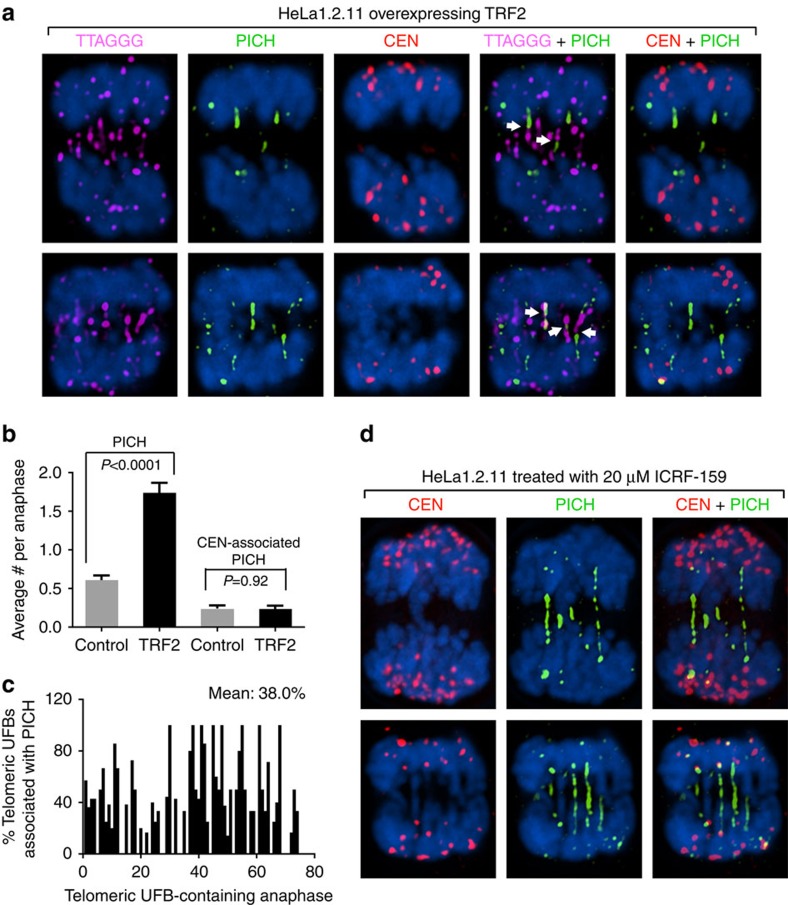
The PICH protein associates with TRF2-induced telomeric UFBs. (**a**) Representative anaphase images showing the staining of PICH, telomeres, and centromeres in HeLa1.2.11 cells overexpressing TRF2. Cells were infected with lentivirus expressing TRF2 and collected at PD2 after infection for immunostaining-FISH analysis. Telomeres (magenta) and centromeres (red) were identified by PNA FISH. PICH (green) were identified by immunostaining with an anti-PICH antibody. Chromosomes were stained with DAPI (blue). PICH-aligned telomeric anaphase bridges were marked by white arrows. Note that the image represents a single section on the *z* axis. (**b**) Quantification of PICH bridges, as well as centromere-associated PICH bridges, in HeLa1.2.11 cells overexpressing GFP control or TRF2. Bars represent mean values and s.e.m. (>150 anaphases from three independent experiments examined for each line). Two-tailed Student's *t*-tests were performed to make pairwise comparison for statistical significance. (**c**) Quantification of fraction of telomeric UFBs that associate with the PICH protein. 75 telomeric UFB-containing anaphases from three independent experiments were examined for the association between PICH and telomeric UFB. (**d**) Representative anaphase images showing the staining of PICH and centromeres in HeLa1.2.11 cells treated with 20 μM DNA topoisomerase II inhibitor ICRF-159.

Immunostaining demonstrated that the characteristic markers of ultrafine anaphase bridges were associated with these TRF2-induced telomeric UFBs. It has been reported that the PICH protein and the BLM helicase colocalize with the centromere- and the CFS-originated UFBs^27^,^28^,^29^,^30^,^31^. A subset of these UFBs, presumably those that have been unwound by BLM, is marked by the single-stranded DNA-binding protein replication protein A (RPA)^46^,^47^. To examine whether the TRF2-induced telomeric anaphase bridges have the above characteristic features of UFBs, we performed immunostaining in TRF2-overexpressing HeLa1.2.11 cells with antibodies against PICH, BLM and RPA proteins followed by FISH analysis using a telomeric probe and a centromeric probe. The PICH and BLM proteins indeed associated with many telomeric UFBs (Fig. 4a, Supplementary Fig. 4a). As shown in Fig. 4b, the average number of centromere-associated UFBs per anaphase did not increase in cells overexpressing TRF2, suggesting that TRF2 overexpression specifically induced the formation of telomeric but not centromeric UFBs. Co-immunostaining of BLM and PICH proteins in cells overexpressing TRF2 showed that PICH and BLM overlapped with each other, forming bridges between the segregating anaphase chromosomes (Supplementary Fig. 4b). Quantification of anaphase bridges in >70 telomeric UFB-containing anaphases showed that ∼38% of the telomeric UFBs colocalized with patches of PICH proteins (Fig. 4c). Interestingly, we note that PICH staining was confined to a segment of the bridge on many telomeric UFBs (Fig. 4a). In contrast, immunostaining of HeLa1.2.11 cells treated with ICRF-159 (a topoisomerase II inhibitor) using antibodies against PICH, followed by centromere FISH analysis, showed that many PICH-positive anaphase bridges formed between the segregating centromeres and that PICH often associates along the entire length of these centromeric UFBs (Fig. 4d), as previously observed by others^30^,^31^. This suggests a possible functional difference of PICH protein on telomeric versus centromeric UFBs, although we cannot rule out the possibility that annealing of the telomeric FISH probe interferes with the detection of PICH protein on telomeric UFBs. We also observed that a subset of the UFBs in cells overexpressing TRF2 was marked by the RPA protein (Supplementary Fig. 4a). BLM and RPA often exhibited an interspersed association pattern along UFBs, suggesting that BLM may dissociate from the unwound DNA strands bound by RPA.

Taken together, our data argue that the telomeric anaphase bridges induced by overexpression of TRF2 are ultrafine anaphase bridges, which arise from persistent replication stalling at telomeres.

### Reduced TRF1 induces fragility but not UFBs at telomeres

Depletion of TRF1 in mouse embryonic fibroblasts has also been reported to cause replication fork stalling and the formation of fragile telomeres^32^,^48^. We therefore examined the possibility that elevated levels of TRF2 may compete with TRF1 for telomere binding, resulting in decreased telomeric TRF1, which leads to telomere replication defects and telomeric UFB formation.

Overexpression of TRF2 indeed significantly decreased the levels of telomere-bound TRF1 (Supplementary Fig. 5a,b). To examine whether the TRF2-induced telomeric UFB formation was simply a secondary consequence of depletion of TRF1, we knocked down TRF1 in HT1080 cells by shRNA treatment (Supplementary Fig. 5c). Bulk telomere length analysis showed that the TRF1 depletion resulted in progressive telomere extension (Supplementary Fig. 5d), in contrast to the stochastic telomere shortening phenotype induced by TRF2 overexpression (Fig. 1c). Furthermore, TRF1 depletion in HT1080 cells led to increased sister telomere associations and fragile telomeres (Supplementary Fig. 5e,f), which is consistent with the TRF1 depletion phenotype previously observed in mouse cells^32^,^48^. Notably, we did not detect any telomeric UFBs in TRF1-depleted HT1080 cells or HeLa1.2.11 cells, among ∼90 anaphases examined for each experiment. These data demonstrate that depletion of TRF1 by itself is not sufficient to induce telomeric UFBs, and also suggest that fragile telomeres may derive from telomere associations that are resolved before cells enter into anaphase.

### UFBs correlate with TRF2-induced telomere shortening

**Figure 5:**
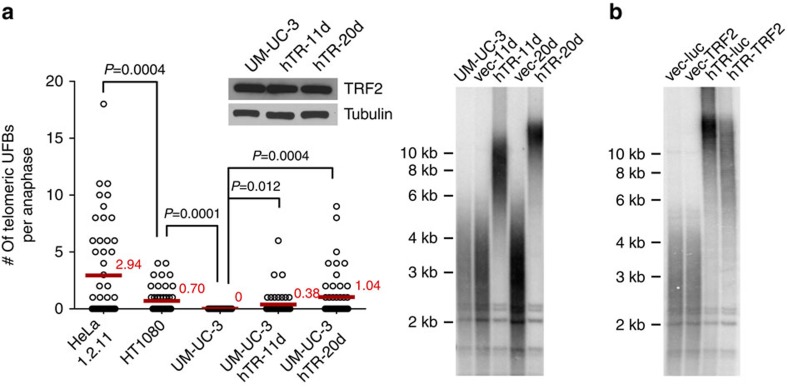
TRF2-induced telomeric UFBs correlate with stochastic telomere shortening. (**a**) Long telomere length exacerbates TRF2-induced telomeric UFBs. Left panel: quantification of telomeric UFBs in cells with different mean telomere lengths. Approximately 50 anaphases from each cell line were analysed for telomeric UFBs. Mean values are indicated in red. Two-tailed Student's *t*-tests were performed to make pairwise comparison for statistical significance. No telomeric UFBs were detected in HeLa1.2.11 and HT1080 cells infected with lentivirus overexpressing GFP. Telomeres in UM-UC-3 cells (∼3 kb) were pre-extended by expression of the telomerase RNA subunit (hTR) for 11 days (to ∼8 kb) and 20 days (to ∼15 kb). Cells were infected with lentivirus expressing TRF2, and then fixed for telomeric FISH 48 h after infection. TRF2 overexpression did not induce any telomeric UFBs in control UM-UC-3 cells that were infected with an empty lentiviral vector. Right panel: terminal restriction fragment analysis in UM-UC-3 cells expressing vector control or hTR using a telomeric repeat probe. (**b**) TRF2 overexpression fails to induce telomere shortening in cells containing very short telomere length.

It is possible that elevated levels of TRF2 lead to the formation of an excess of tight DNA–protein complexes, which impede replication fork progression at telomeres. This model predicts that longer telomeres containing more TRF2-binding sites would exacerbate TRF2-induced UFBs. To examine whether TRF2-induced telomeric UFB formation correlated with telomere lengths, we compared the induction of telomeric UFB in cells containing different mean telomere lengths: HeLa 1.2.11 ∼20 kb; HT1080 A6 ∼8 kb; and UM-UC-3 ∼3 kb. As shown in Fig. 5a, comparable levels of TRF2 induced significantly more telomeric UFBs in cells with longer telomeres. Interestingly, in UM-UC-3 cells containing very short telomeres (mean length ∼3 kb), TRF2 overexpression did not induce any telomeric UFBs. To determine whether short telomere length was the sole reason responsible for the failure to induce telomeric UFBs in UM-UC-3 cells, telomeres were elongated by overexpressing the telomerase RNA subunit (Fig. 5a), and examined for the induction of telomeric UFBs. Although TRF2 overexpression failed to induce telomeric UFBs in parental UM-UC-3 cells or in cells expressing an empty vector, comparable levels of TRF2 expression induced significant numbers of telomeric UFBs in UM-UC-3 cells containing pre-extended telomeres (Fig. 5a). Bulk telomere-length analysis conducted seven population doublings after TRF2 overexpression showed drastic and rapid telomere shortening in UM-UC-3 cells containing pre-extended telomeres, but not in control cells whose telomeres are not pre-extended (Fig. 5b). These data demonstrate that the TRF2-induced telomere shortening closely correlated with the formation of telomeric UFBs, suggesting that the shortening of telomeres result from cells' resolution of telomeric UFBs.

## Discussion

In this study, we have elucidated the molecular series of events that occur at chromosome ends in response to elevated levels of TRF2. By examining the length of individual telomeres in cells overexpressing TRF2, we uncovered a subpopulation of termini that had undergone loss of almost the entire telomeric tract, which was often accompanied by end-to-end fusions. Our data also demonstrate that persistent replication stalling was induced by TRF2 overexpression, resulting in the formation of UFBs during the subsequent anaphase. Strikingly, telomeric UFBs between segregating anaphase chromosomes could be observed as early as the first cell division after TRF2 overexpression, before detection of significant telomere shortening (which required at least three to four cell divisions after TRF2 overexpression). These data support a model in which the primary defect caused by TRF2 overexpression is inhibition of duplex telomeric DNA replication, with resolution of the resulting UFBs leading to stochastic loss of large segments of telomeric sequences.

Our observations therefore provide a second mechanism by which perturbation of normal TRF2 levels can influence genomic instability. Experimental removal of TRF2 from telomeres causes chromosome end-to-end fusions, which often preserve long tracts of telomeric repeats on either side of the fusion junction^49^,^50^,^51^. In contrast, we found that the majority of the TRF2 overexpression-induced chromosome fusions were accompanied by extensive deletions into the subtelomeric regions of involved chromosomes. Furthermore, these fusion junctions often contained one to six nucleotides of microhomology between the fused chromosomes. Fusions of similar features have been detected in human and mouse cells containing critically shortened telomeres, as well as in early-stage colon carcinoma and chronical lymphocytic leukaemia cells^37^,^38^,^39^,^40^,^41^. Critically shortened telomeres are known to be fused by the alternative non-homologous end-joining (A-NHEJ) repair process^52^. Extensive deletions and limited microhomology at the fusion junctions are among the characteristic features of A-NHEJ^53^,^54^. Future studies are needed to determine the involvement of A-NHEJ in TRF2 overexpression-induced fusions. Since chromosome fusions can inflict genomic instability, it will also be important to examine TRF2 levels in staged tumour samples and determine whether dysregulation of TRF2 correlates with tumorigenic transformation.

The specialized telomeric DNA structures at mammalian chromosome ends impose great challenges for replication: the single-stranded telomeric 5′-TTAGGG-3′ repeats exposed during replication can form G-quadruplex structures, which hinder lagging-strand replication; the T-loop structures formed by invasion of the 3′-single-stranded telomeric overhang into the duplex region of telomeres present topological barriers for telomere replication. Shelterin protein TRF1 and multiple helicases (that is, BLM, RTEL1 and WRN) are implicated in the removal of these replication blockades so that replication forks can progress smoothly at telomeres^32^,^55^,^56^,^57^,^58^,^59^. Although TRF2 overexpression caused a reduction of telomeric TRF1, knocking down of TRF1 did not induce telomeric UFBs, even though it resulted in significant telomere fragility. The observation that TRF1 depletion led to telomere elongation but not telomere rapid deletions strongly suggests that the stalled replication forks caused by TRF1 depletion were resolved differently from those caused by TRF2 overexpression. The amount of TRF2-induced telomeric UFBs per cell increased significantly when telomeres were extended by telomerase, suggesting that the majority of telomeric UFBs were formed by terminal—but not interstitial—telomeric sequences. It has been reported previously that cells depleted of TRF1 by siRNA treatment or cells deficient for WRN contain BLM-associated UFBs extending from one or two telomeric foci^56^. Nonetheless, such UFBs do not seem to be of telomeric sequences since they do not hybridize to a telomere repeat probe in telomeric FISH analysis. The genomic sequences from which these UFBs originate remain to be determined.

Elevated levels of TRF2 might lead to the formation of an excess of tight DNA–protein complexes, which exhaust the cellular regulatory system that remove them during replication under normal conditions, thus stall replication at telomeres. In fact, purified recombinant TRF1 and TRF2 proteins have been observed to stall replication fork progression at telomeric DNA in an *in vitro* SV40-based replication system^26^. Curiously, the RTEL1 helicase interacts with TRF2 (ref. 60) and knockout of RTEL1 in mouse embryonic fibroblasts was found to cause telomere fragility and stochastic deletion of telomeric tracts^55^, phenocopying the consequences of TRF2 overexpression. Although we failed to detect any telomeric phenotypes by knocking down RTEL1 to ∼30% of the endogenous levels in HeLa1.2.11 cells, it is premature to exclude the involvement of RTEL1 in telomeric UFB formation/resolution since the residual RTEL1 in cells may be sufficient to carry out its telomeric functions. A recent live microscopy study of TRF1 overexpression in mouse embryonic stem cells demonstrated that very high TRF1 levels resulted in telomere associations that later became anaphase telomeric bridges and interphase telomere aggregates^61^. It will be interesting to examine whether TRF1 overexpression causes the same type of telomeric UFBs as TRF2 overexpression.

It is noteworthy that the PICH protein often associates along a segment of the TRF2-induced telomeric UFBs, but along the entire length of the centromere- or CFS-originated UFBs (Fig. 4, Supplementary Fig. 4)^27^,^30^,^31^. Although we cannot exclude the possibility that annealing of the telomeric FISH probe interferes with detection of PICH at telomeric UFBs, this difference might be due to the unique nature of telomere replication: First, unlike CFS where opposing replication forks converge, at telomeric sequences the replication fork progresses largely unidirectionally^32^,^43^ from the subtelomeric region toward the end of the chromosome. The stalled unidirectional, non-converging replication fork could be processed differently from the converging replication forks. Second, the association and dissociation of PICH and BLM with telomeric tracts might be influenced by the shelterin protein complexes: for example, both the duplex telomeric DNA-binding protein TRF2 and the single-stranded telomeric DNA-binding protein POT1 have been reported to interact with BLM and stimulate its helicase activity^62^,^63^,^64^,^65^. Last, other helicases (that is, RTEL1 and WRN) in addition to BLM are known to facilitate telomere replication^56^,^58^,^66^, therefore, they may also be involved in resolving TRF2-induced telomeric UFBs.

## Methods

### Cell lines

HT1080 fibrosarcoma cells, HeLa cervical cancer cells, UM-UC-3 urinary bladder cancer cells, breast cancer cell lines MDA-MB-231, MDA-MB-453, MDA-MB-468, ZR-75-1, MCF7, SK-BR-3, human primary fibroblast cell lines IMR90, BJ and WI38 were obtained from ATCC. Melanoma cell lines WM115, WM278, WM1158, WM983A and WM983B were obtained from the Melanoma Cell Line Repository at the Wistar Institute. All cell lines from ATCC and the Melanoma cell line repository have been verified by STR profiling and tested for Mycoplasma by the distributors. CaCL 73-36 melanoma cell line^67^ was kindly provided by Dr Donna George at University of Pennsylvania. LOX melanoma cell line^68^ was kindly provided by Dr Mohammed Kashani-Sabet at University of California, San Francisco. LOX melanoma cells were grown in RPMI-1640 supplemented with 10% fetal bovine serum. All other cancer cell lines were grown in DMEM supplemented with 10% fetal bovine serum, and primary cell lines in DMEM supplemented with 15% fetal bovine serum.

### Immunoblotting analysis

Whole-cell extracts were resolved with 10% SDS–PAGE and transferred to PVDG nitrocellulose membranes. Immunoblots were incubated with a mouse monoclonal anti-TRF2 (BD Transduction Laboratories, 1:500), followed by a horseradish peroxidase-conjugated donkey anti-mouse IgG (Jackson ImmunResearch). ECL Prime reagent (GE Healthcare) was used for signal detection. The same blot was stripped and reprobed with a mouse monoclonal anti-tubulin antibody (Sigma-Aldrich) as loading controls. Full scans of western blots are provided in Supplementary Fig. 6.

### Lentiviral plasmids

The pHR'CMV lentiviral expression vector system used in this study was provided by Dr Didier Trono. TRF2 expression lentiviral vector contains the full-length, untagged, wild-type TRF2 cDNA driven by the CMV promoter, followed by an internal ribosome entry site and a hygromycin resistance gene. The GFP-TRF2 expression lentiviral vector used in Supplementary Fig. 5 contains an N-terminal GFP-tagged TRF2 cDNA. Telomerase RNA expression lentiviral vector contain the wild-type hTR cDNA driven by the IU1 promoter and a GFP gene driven by the CMV promoter^69^. The shRNA expression lentiviral vector was constructed as described previously^69^. The target sequence for TRF1 shRNA is 5′-GGAACATGACAAACTTCATGA-3′.

### Terminal restriction fragment analysis

Five microgram of genomic DNA was digested with Hinf I and Rsa I, fractionated by 0.6% agarose-TBE gel electrophoresis, and transferred to a Hybond XL membrane. Southern blotting was carried out with an end-labelled telomeric probe (C_3_TA_2_)_4_. Blots were analysed by the ImageQuant software. Mean telomere lengths were calculated according to the positions of molecular weight markers run on the same gel.

### Single telomere length analysis

Briefly, 20 ng EcoRI-digested genomic DNA was incubated in a 10-μl ligation reaction containing 0.9 μM anchor oligo and 1 U T4 DNA ligase (Roche) in 1 × manufacturer's ligation buffer at 35 °C for 12 h. The ligated DNA was diluted to 50 pg μl^−1^ for subsequent multiple PCRs. Each PCR (94 °C for 2 min, 25 cycles of 94 °C for 15 s, 65 °C for 30 s, and 68 °C for 10 min followed by a final extension step at 68 °C for 20 min) was carried out in a 15-μl reaction volume containing 100 pg of ligated DNA, 0.5 μM each primer, 0.3 mM each dNTP, 75 mM Tris-HCl (pH8.8), 20 mM (NH4)_2_SO_4_, 0.01% Tween-20, 1.5 mM MgCl_2_ and 1.5 U Extensor Hi-Fidelity PCR Enzyme Mix (Abgene). PCR products were resolved on 0.6% agarose gels and transferred onto Hybond XL membrane (GE Healthcare), followed by hybridization with a subtelomeric probe generated by PCR using primer pair XpYpE2 and XpYpB2. Signals were detected by phosphorimaging (Molecular Dynamics). Sequence of oligonucleotides used: anchor oligo, 5′-TGCTCCGTGCATCTGGCATCCCTAACC-3′; PCR primer 1, 5′-TGCTCCGTGCATCTGGCATC-3′; PCR primer 2 (XpYpE2), 5′-GTTGTCTCAGGGTCCTAGTG-3′; XpYpB2, 5′-TCTGAAAGTGGACC(A/T)ATCAG-3′.

### Fusion PCR

Briefly, genomic DNA was extracted using Gentra Puregene Cell kit (Qiagen) and diluted to 20 ng μl^−1^ in 10 mM Tris-HCl (pH 7.5). Each PCR (94 °C for 2 min, 25 cycles of 94 °C for 15 s, 59 °C for 30 s, and 68 °C for 10 min followed by a final extension step at 68 °C for 20 min) was carried out in a 15-μl reaction volume containing 100 ng of genomic DNA, 0.5 μM each of telomere-adjacent primers (XpYpM, 17p6 and 21q1), 0.3 mM each dNTP, 75 mM Tris-HCl (pH8.8), 20 mM (NH4)_2_SO_4_, 0.01% Tween-20, 1.5 mM MgCl_2_ and 1.5 U Extensor Hi-Fidelity PCR Enzyme Mix (Abgene). PCR products were resolved on 0.6% agarose gels and transferred onto Hybond XL membrane (GE Healthcare), followed by hybridization with a subtelomeric probe generated by PCR using primer pair XpYpO and XpYpB2.

For sequencing analysis of fusion molecules, fusion PCR products were reamplified: First-round PCR products were diluted 1:20 in H_2_O, and 3 μl were used in second-round PCR with telomere adjacent primers XpYpO, 17p7 and 21qseq1 under the same PCR condition except with 3 mM MgCl_2_ and amplified for 32 cycles. Reamplified DNA was gel purified and sequenced with XpYp and 17p subtelomeric primers. Sequence of oligonucleotides used: XpYpM, 5′-ACCAG GTTTTCCAGTGTGTT-3′; XpYpO, 5′-CCTGTAACGCTGTTAGGTAC-3′; 17p6, 5′-GGCTGAACTATAGCCTCTGC-3′; 17p7, 5′-CCTGGCATGGTATTGACATG-3′; 21q1, 5′-CTTGGTGTCGAGAGAGGTAG-3′; 21qseq1, 5′-TGGTCTTATACACTGTGTTC-3′.

### Metaphase fluorescence *in situ* hybridization

Metaphase fluorescence in situ hybridization was performed using an Alexa488-OO-5′-(CCCTAA)_3_-3′ (telomeric sequence) and a TMR-OO-5′-CTTCGTTGGAAACGGGA-3′ (centromeric sequence) PNA probe (Panagene). Images were acquired with a Nikon Ti-U microscope using a × 60 objective. All image files were mixed and randomly assigned coded names to allow blinded scoring for chromosome fusions, signal-free ends and fragile telomeres.

### Immunofluorescence staining and FISH

Cells grown on coverslips were fixed with 2% paraformaldehyde and permeabilized with 0.5% NP-40. Immunofluorescence staining was carried out by incubating with an anti-BLM antibody (Santa Cruz Biotechnology, sc-7790, 1:150), an anti-RPA34 antibody (GeneTex, clone 9H8, 1:500), an anti-PICH antibody (Abnova, H54821-B01P, 1:500), or an anti-TRF1 antibody (GeneTex, clone 4E4, 1:500), followed by secondary antibody conjugated with respective Alexa Fluorophores (Molecular Probes, 1:500). The cells were fixed again with 4% paraformaldehyde and dehydrated by successive incubation in 70, 95 and 100% ethanol before subjected to FISH analysis. PNA probes for FISH analysis were TMR-, Cy5- or Alexa488-OO-5′-(CCCTAA)_3_-3′ (telomeric sequence). DNA was stained by 0.1 μg ml^−1^ DAPI. Coverslips were mounted onto glass slides in Prolong Gold Antifade Reagent (Invitrogen). Images were acquired with a Nikon Ti-U microscope using a × 100 objective and collected as a stack of 0.2-μm increments in the *z* axis. Image deconvolution was conducted using the AutoQuant X3 software. Unless otherwise noted, images were viewed as a single section on the *z* axis.

### Chromatin fibre FISH analysis

Asynchronous populations of cells were first labelled with 30 μM of IdU for 4 h, washed three times with PBS and then labelled with 30 μM CldU for another 4 h. Chromatin fibres were prepared as described in ref. 70. Briefly, cells were trypsinized, hypotonically treated in 0.5% sodium citrate and cytospinned onto superfrost plus glass slides. Slides were dipped into lysis buffer (10 mM Tris pH 7.5, 1% Triton X-100 and 500 mM Urea) and incubated for 20 min. Chromatin was stretched by slowly removing slides vertically from the lysis buffer and fixed by incubating in methanol supplemented with 0.1% β-mercaptoethanol. Stretched chromatin was denatured in alkali buffer (0.1 M NaOH, 70% ethanol, and 0.1% β-mercaptoethanol), fixed in alkali buffer supplemented with 0.5% glutaraldehyde and dehydrated by successive incubation in 70, 90 and 100% ethanol. Telomeric DNA was detected by hybridization with a 5′-Biotin-OO-(CCCTAA)_3_-3′ PNA probe, followed by sequential incubation with Alexa568-conjugated streptavidin (Molecular Probes, 1:1,000), biotinylated anti-streptavidin antibody (Vector, 1:250), and then Alexa568-conjugated streptavidin. IdU and CldU were detected with a mouse anti-IdU antibody (Becton Dickinson, B44, 1:10) and a rat anti-CldU antibody (AbD Serotec, BU1/75, 1:40), followed by incubation with Alexa 350-conjugated goat anti-mouse and Alexa 488-conjugated goat anti-rat secondary antibodies (Molecular Probes, 1:100). Images were acquired with a Nikon Ti-U microscope using a × 60 objective. Individual slides were blinded before image acquisition to avoid bias in the analysis.

### Real-time PCR

Total RNA was extracted with the TRIzol reagent (Invitrogen). cDNA was prepared using the High Capacity RNA-to-cDNA kit (Invitrogen). Real-time PCR was performed using the Power SYBR green PCR master mix on the StepOnePlus real-time PCR machine (Invitrogen). Telomerase RNA levels were normalized against GAPDH mRNA levels. Primer sets used: TRF1 forward 5′-CGCAACAGCGCAGAGGCTATTATT-3′, TRF1 reverse 5′-ATCATCAGGGCTGATTCCAAGGGT-3′; GAPDH forward 5′-CATGTTCGTCATGGGTGTGAACCA-3′, GAPDH reverse 5′-ATGGCATGGACTGTGGTCATGAGT-3′.

## Additional information

**How to cite this article:** Nera, B. *et al.* Elevated levels of TRF2 induce telomeric ultrafine anaphase bridges and rapid telomere deletions. *Nat. Commun.* 6:10132 doi: 10.1038/ncomms10132 (2015).

## Supplementary Material

Supplementary Information Supplementary Figures 1-6
