# Supplementary material for: Elevated levels of TRF2 induce telomeric ultrafine anaphase bridges and rapid telomere deletions
Source: Nat Commun. 2015 Dec 7;6:10132. doi: 10.1038/ncomms10132 (PMC4686832; doi:10.1038/ncomms10132)
Supplement: Supplementary Information — Supplementary Figures 1-6 [file ncomms10132-s1.pdf]

Supplementary Figures

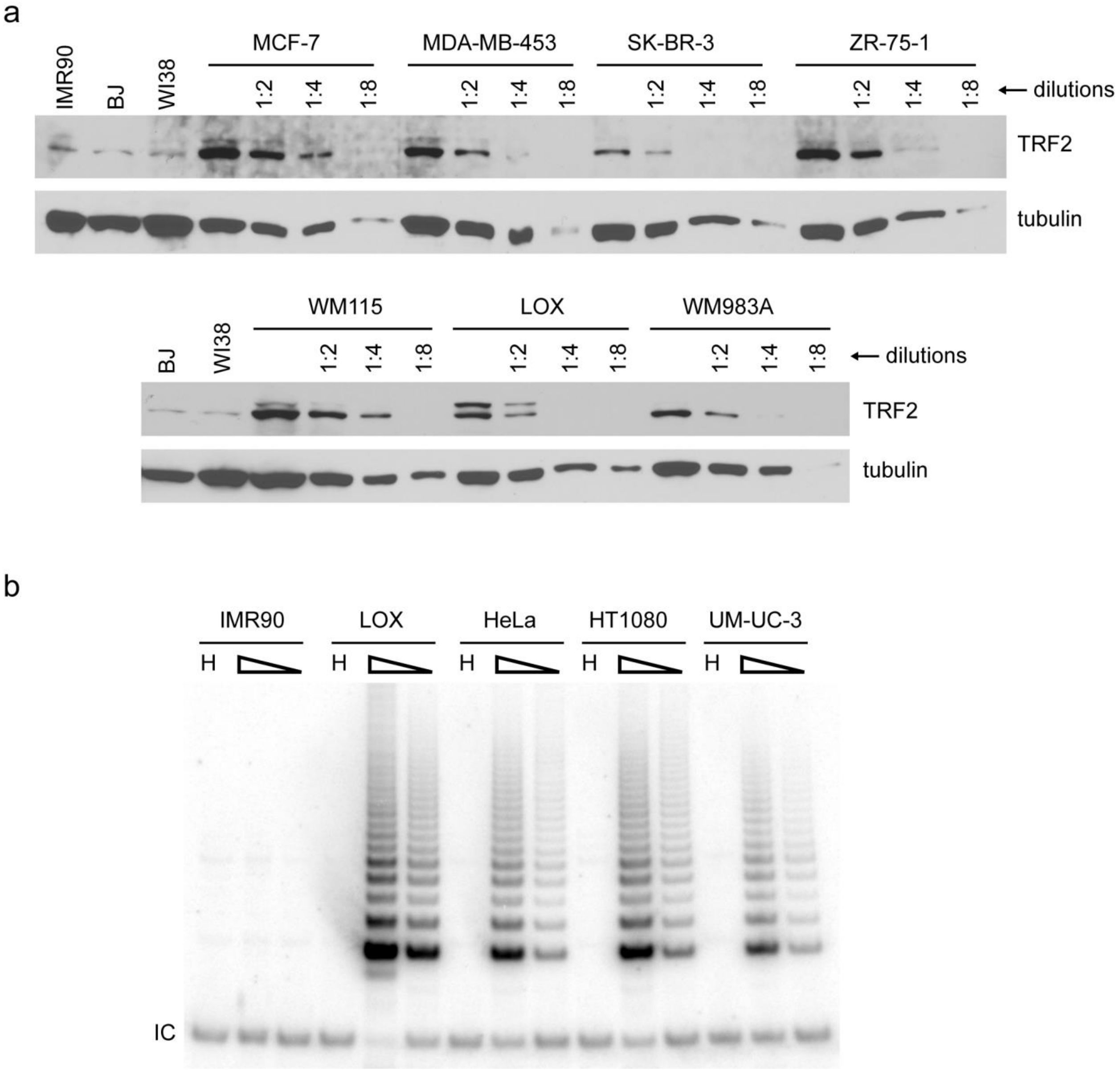

Supplementary Figure 1. Elevated levels of TRF2 in breast cancer and melanoma cells. (**a**) Assessing relative TRF2 protein levels by immunoblotting analysis of a serial dilution of whole cell extracts from different cell lines. Primary fibroblasts: IMR90, BJ, and WI38; Breast cancer cells: MCF-7, MDA-MB-453, SK-BR-3, ZR-75-1; Melanoma cells: WM115, LOX, and WM983A. Tubulin was used as a loading control. (**b**) Telomerase activity measured by TRAP assay. The triangles at the top indicate sequential fourfold dilutions of whole cell extracts starting at 20ng whole cell extracts per assay. The internal control (IC) is used to normalize PCR activity. H: extract treated by heat before subjecting to TRAP analysis.

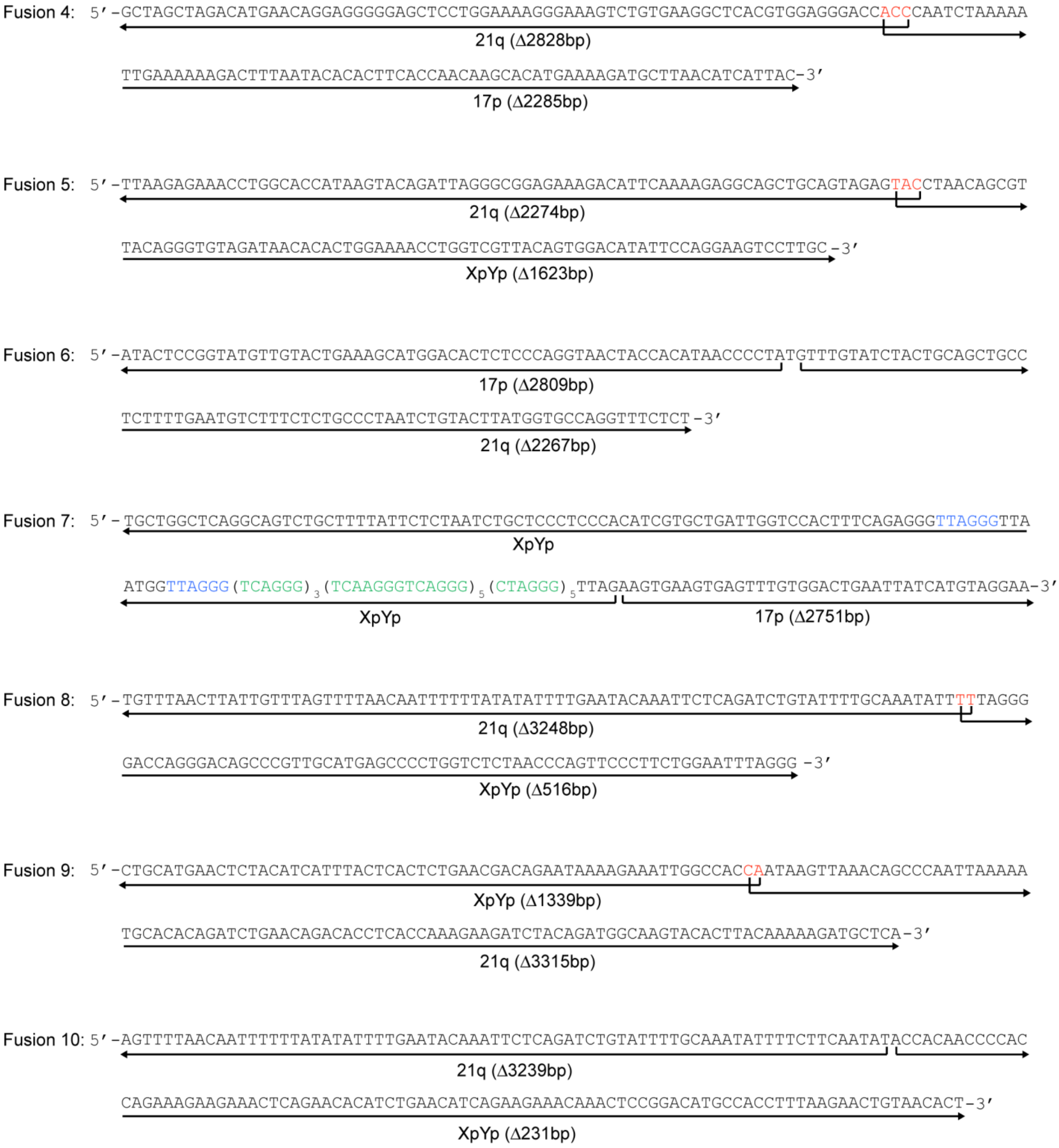

Supplementary Figure 2. Additional sequences of fusion molecules induced by TRF2 overexpression in HeLa1.2.11 cells. Cells infected with lentivirus overexpressing TRF2 were harvested at PD6. Multiple aliquots of 100ng of genomic DNA were subjected to fusion PCR using a mix of XpYp, 17p and 21q subtelomeric primers. PCR products were sequenced using additional XpYp, 17p and 21q subtelomeric primers as explained in Methods. The fusion points, size of deletions, and microhomology (in red) are indicated. Note that only more than three contiguous TTAGGG repeats were deemed as telomeric repeats. Fusion 7 contains telomeric variant repeats (in green) and two non-contiguous TTAGGG sequence (in blue).

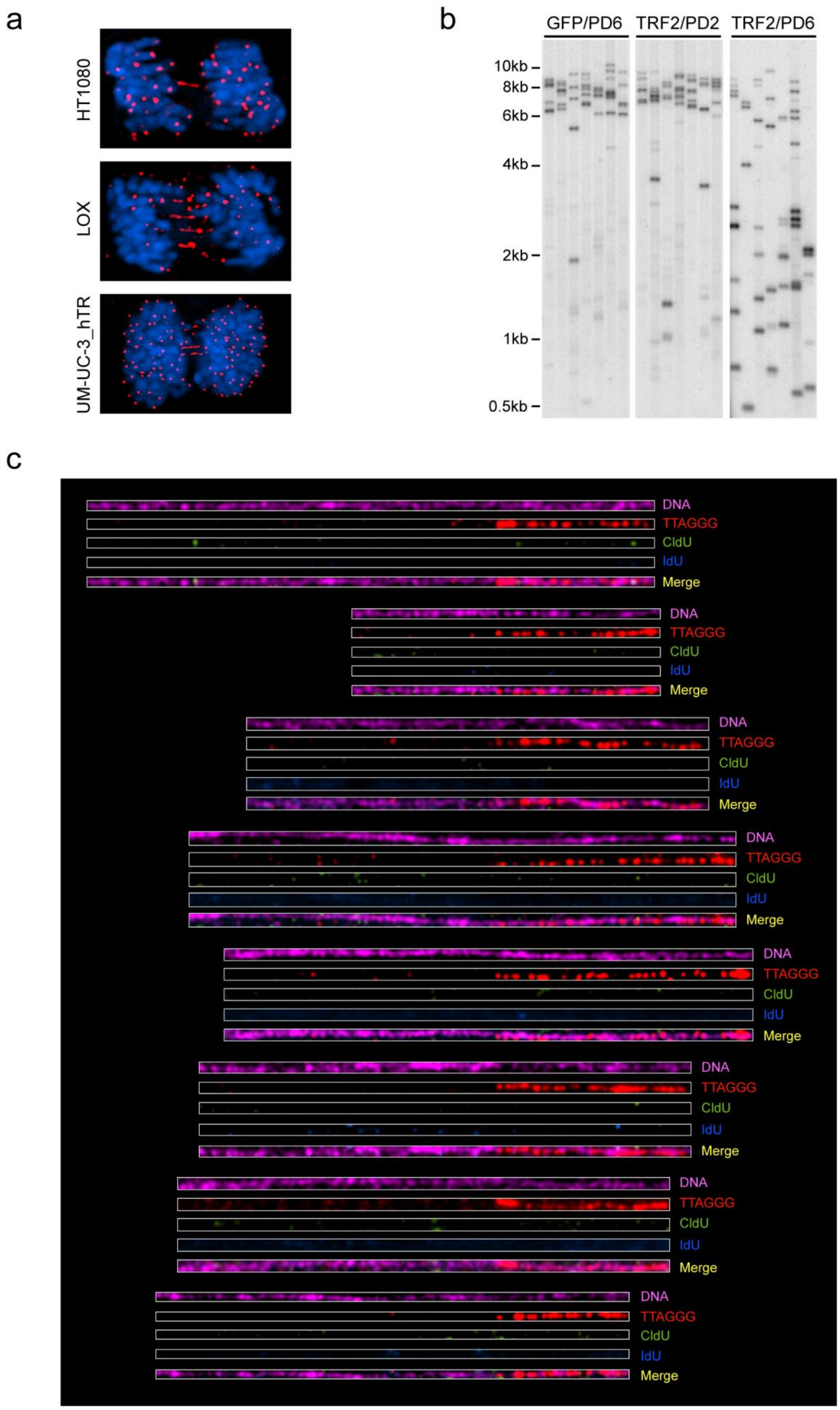
a
HT1080
LOX
UM-UC-3_hTR
b
GFP/PD6
TRF2/PD2
TRF2/PD6
10kb
8kb
6kb
4kb
2kb
1kb
0.5kb
c
DNA
TTAGGG
CldU
IdU
Merge

Supplementary Figure 3. TRF2 overexpression induced telomeric UFBs. (**a**) Overexpression of TRF2 induced telomere bridges between the segregating anaphase chromosomes in HT1080, LOX, and UM-UC-3 hTR-11d cells. Telomeric DNAs were detected by *in situ* hybridization with a PNA telomeric probe (red). Chromosomes were stained with DAPI (blue). (**b**) Individual telomere lengths measured by STELA analysis in HT1080 A6 cells overexpressing GFP at PD6, TRF2 at PD2 and PD6. Each lane represents a single PCR reaction performed with 100pg of genomic DNA, followed by Southern blotting detection of XpYp telomeres using an XpYp subtelomeric probe. (**c**) DNA polymerase inhibitor aphidicolin stalled replication in LOX cells. Cells were treated with 1μg/ml aphidicolin for 16 hours and then labeled sequentially with IdU and CldU for 4 hours each in the presence of aphidicolin. Chromatin Fiber FISH analysis was carried out. Telomeres (red) were identified by FISH with a telomeric repeat probe. IdU (green) and CldU (blue) were identified by immunostaining with analog-specific antibodies. To aid the visualization of telomere-adjacent subtelomeric regions, we stained genomic DNA (magenta) with an anti-ssDNA antibody (1:150, MAB3034; Millipore). Note the lack of IdU and CldU incorporation in both the telomeric and subtelomeric regions in aphidicolin-treated cells. This is different from the TRF2-induced replication stalling at the boundary between telomeric and subtelomeric regions.

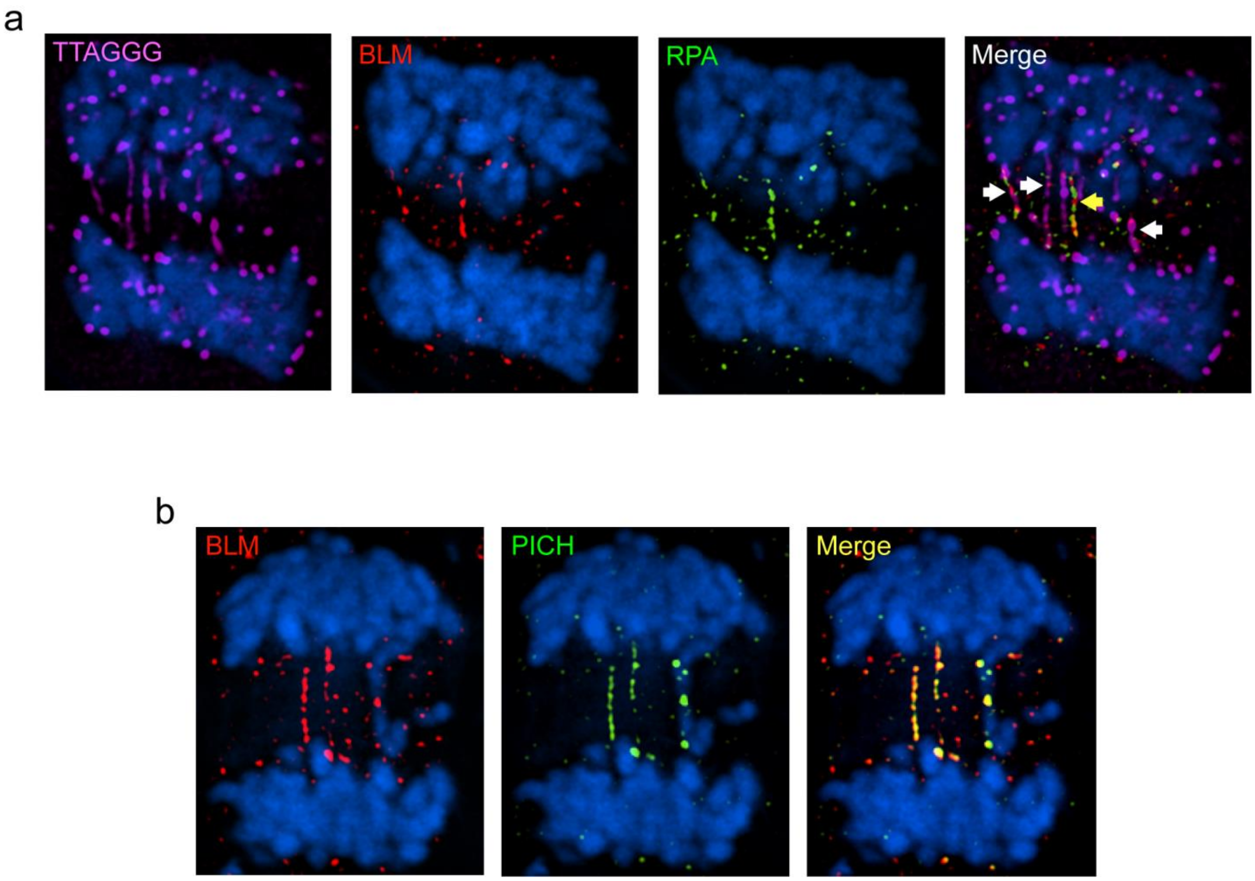

Supplementary Figure 4. TRF2 overexpression induced the formation of PICH- and BLM-associated telomeric UFBs. (**a**) Representative image of anaphase cells showing that the BLM and RPA proteins associate with the TRF2-induced telomeric UFBs. Telomeres (magenta) were identified by PNA FISH. BLM (red) and RPA (green) were identified by immunostaining with an anti-BLM antibody and an anti-RPA antibody. BLM aligned non-telomeric UFBs were marked by a yellow arrow. BLM aligned telomeric anaphase bridges were marked by white arrows. Note that the image represents a single section on the z-axis. (**b**) Representative image showing that the PICH and BLM proteins colocalize to anaphase bridges in cells overexpressing TRF2. HeLa1.2.11 cells were infected with lentivirus overexpressing TRF2. Immunostaining was performed at PD2 with an anti-BLM antibody (red) and an anti-PICH antibody (green).

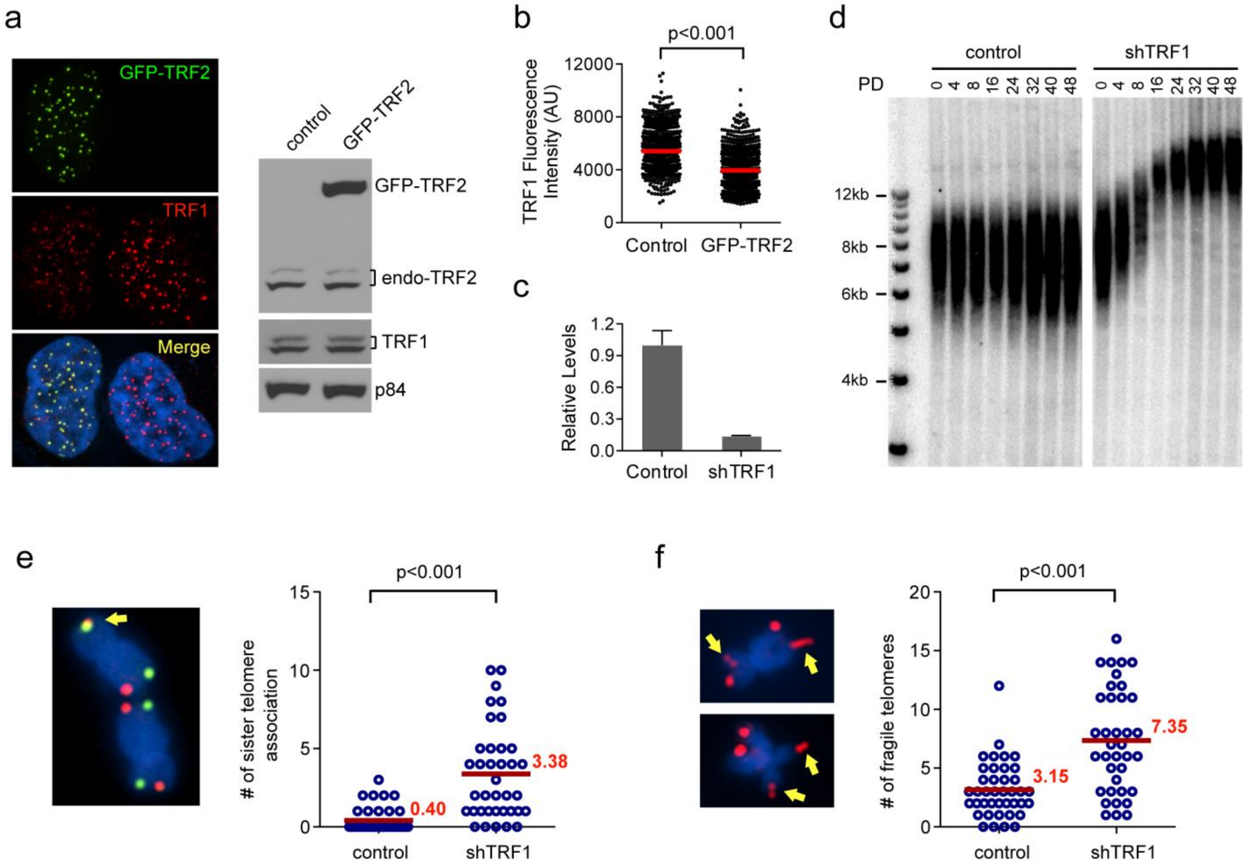

Supplementary Figure 5. Telomeric changes induced by depletion of TRF1. (**a**) TRF2 overexpression delocalizes TRF1 from telomeres. HeLa1.2.11 cells were infected with lentiviruses expressing GFP-TRF2 at a sub-saturating MOI (~70% cells infected). Left panel: immunostaining showing that the infected cell expressing GFP-TRF2 had decreased level of telomeric TRF1 (red) than the uninfected cell on the right. Right panel: immunoblotting of nuclear extracts showing that the expression of GFP-TRF2 did not change the overall cellular level of TRF1. P84 nuclear protein was used as a loading control. (**b**) Scatter plot showing decreased fluorescence intensity (in arbitrary units) of telomeric TRF1 in cells expressing GFP-TRF2 as described in (**a**). Uninfected cells were used as controls. Fluorescence intensity of telomeric TRF1 spots in 30 each of infected and uninfected cells was quantified using the NIS-Elements AR software. (**c**) Relative levels of TRF1 mRNA determined by QPCR in HT1080 A6 cells treated with lentivirus expressing a vector control or a TRF1-targeting shRNA. Bars represent mean values of three experiments and SDs. (**d**) Bulk telomere lengths of HT1080 A6 cells determined by Southern blotting analysis using a telomeric repeat probe. (**e**) Quantification of sister telomere associations. Metaphase spreading followed by Chromosome Orientation FISH (CO-FISH) was carried out to determine sister telomere associations. The CO-FISH image on the left panel shows a representative sister telomere association. (**f**) Quantification of fragile telomeres. Metaphase spreading followed by telomeric FISH was carried out to examine fragile telomeres. Representative fragile telomeres are marked by yellow arrows on images at the left panel. All quantifications in (**e**) and (**f**) were carried out blindly. Note that we did not detect any telomeric UFBs in ~90 anaphases from shTRF1-treated HeLa1.2.11 or HT1080 A6 cells.

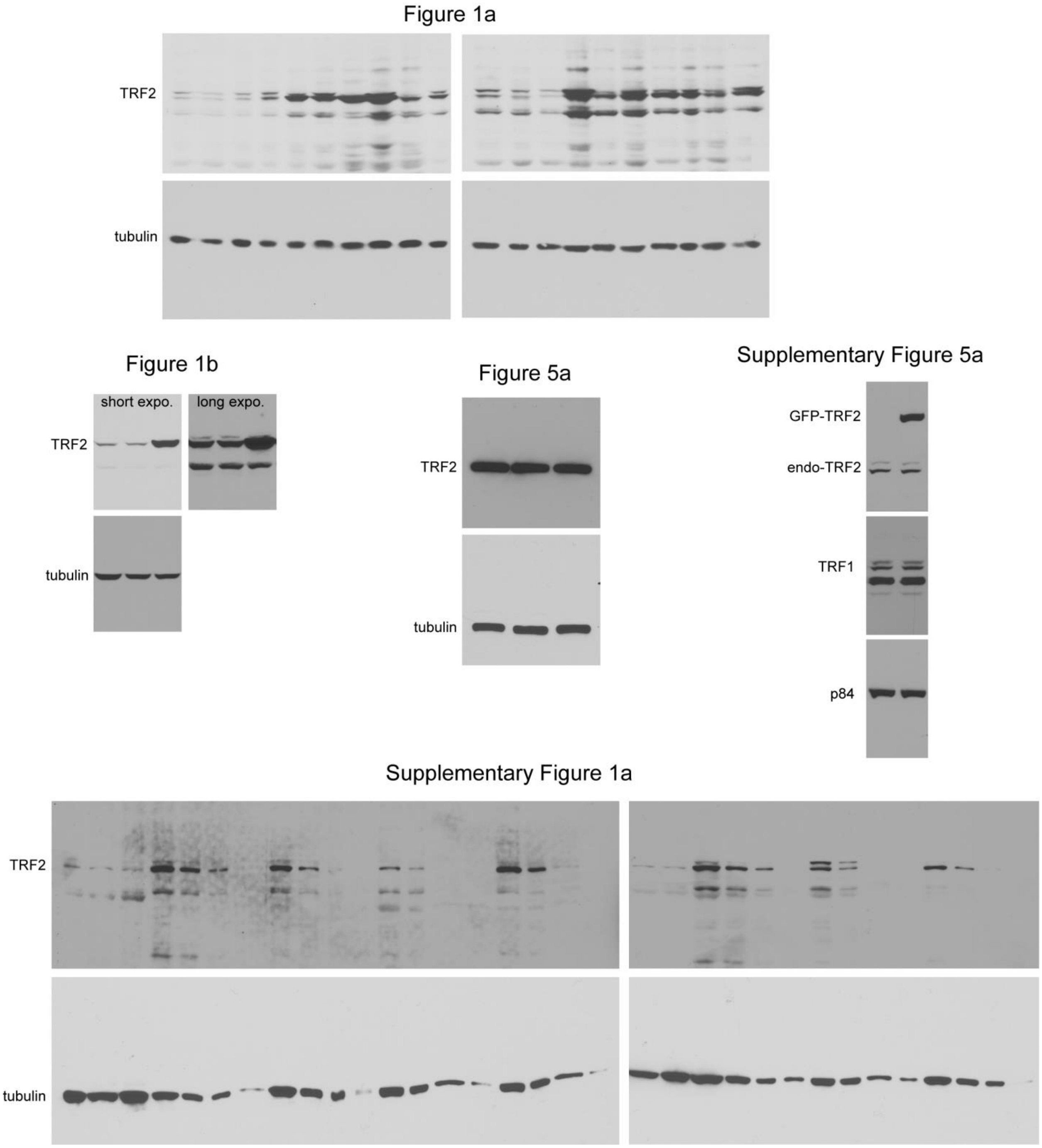

Supplementary Figure 6. Full scans of Western blots.
